# Micromanaging Immunity in the Murine Host vs. the Mosquito Vector: Microbiota-Dependent Immune Responses to Intestinal Parasites

**DOI:** 10.3389/fcimb.2018.00308

**Published:** 2018-09-03

**Authors:** Ivet A. Yordanova, Suzana Zakovic, Sebastian Rausch, Giulia Costa, Elena Levashina, Susanne Hartmann

**Affiliations:** ^1^Center for Infection Medicine, Institute of Immunology, Freie Universität Berlin, Berlin, Germany; ^2^Vector Biology Unit, Max Planck Institute for Infection Biology, Berlin, Germany

**Keywords:** microbiota, gastrointestinal parasite, immune response, mammalian host, mosquito vector, probiotics, *Plasmodium*

## Abstract

The digestive tract plays a central role in nutrient acquisition and harbors a vast and intricate community of bacteria, fungi, viruses and parasites, collectively known as the microbiota. In recent years, there has been increasing recognition of the complex and highly contextual involvement of this microbiota in the induction and education of host innate and adaptive immune responses under homeostasis, during infection and inflammation. The gut passage and colonization by unicellular and multicellular parasite species present an immense challenge to the host immune system and to the microbial communities that provide vital support for its proper functioning. In mammals, parasitic nematodes induce distinct shifts in the intestinal microbial composition. Vice versa, the commensal microbiota has been shown to serve as a molecular adjuvant and immunomodulator during intestinal parasite infections. Moreover, similar interactions occur within insect vectors of deadly human pathogens. The gut microbiota has emerged as a crucial factor affecting vector competence in *Anopheles* mosquitoes, where it modulates outcomes of infections with malaria parasites. In this review, we discuss currently known involvements of the host microbiota in the instruction, support or suppression of host immune responses to gastrointestinal nematodes and protozoan parasites in mice, as well as in the malaria mosquito vector. A deeper understanding of the mechanisms underlying microbiota-dependent modulation of host and vector immunity against parasites in mammals and mosquitoes is key to a better understanding of the host-parasite relationships and the identification of more efficient approaches for intervention and treatment of parasite infections of both clinical and veterinary importance.

## Introduction

**Figure 1:**
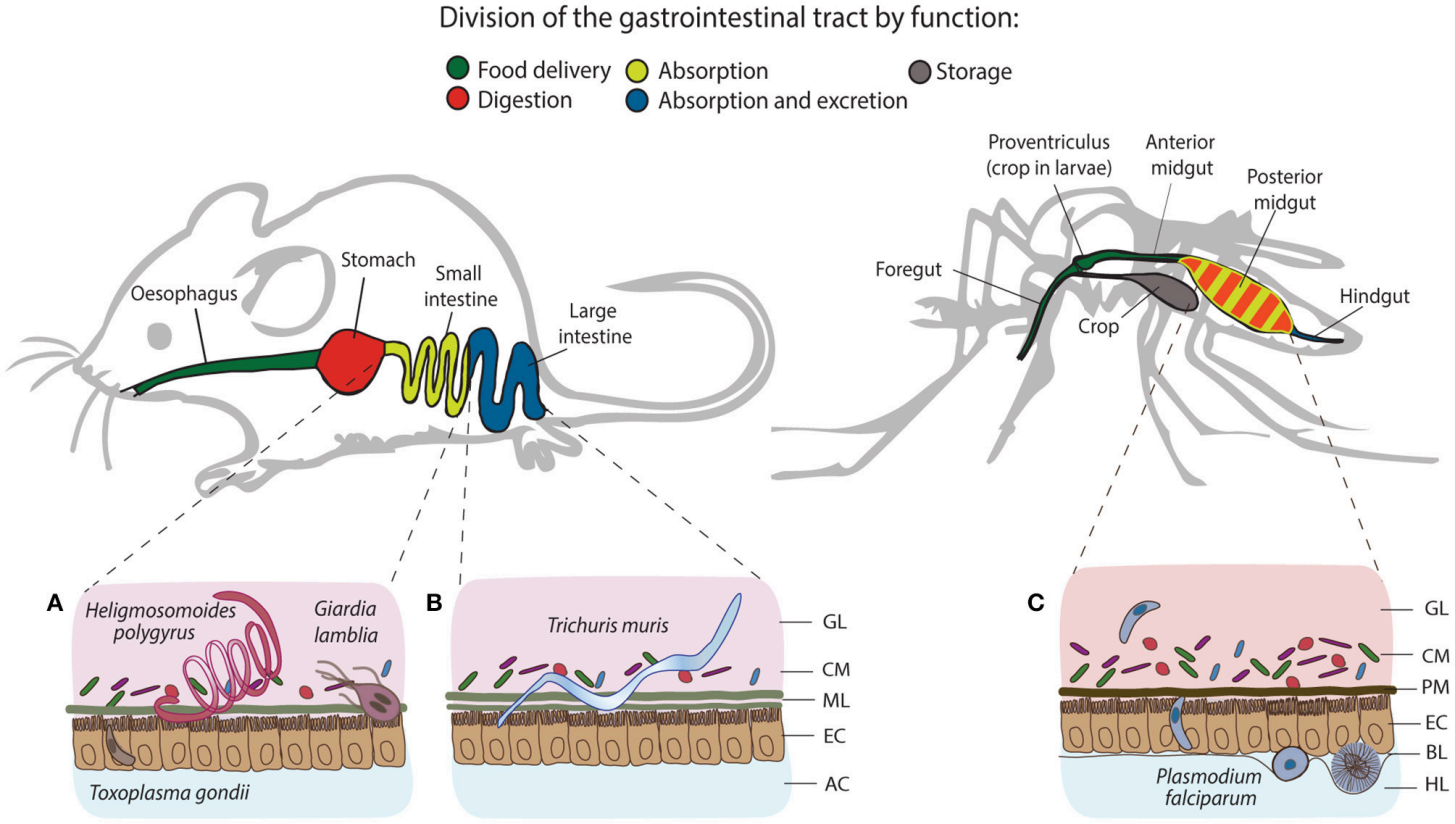
Structural and functional differences of the gastrointestinal tracts of mice and mosquitoes, and the localization of their intestinal parasites. The gastrointestinal tract of mice consists of esophagus, stomach, small and large intestine, and colon, whereas in mosquitoes, it comprises the foregut, diverticulum (crop), midgut (anterior and posterior) and hindgut. One of the differences between gut organization is the separation of the food digestive and nutrient absorption functions taking place in the stomach and the small intestine of mice. In mosquitoes, digestion and absorption occur in the same organ, the midgut. The tracts secrete a protective layer that serves as a mechanical barrier against the acidic gastric environment and diverse pathogens. In mammals, epithelial goblet cells dispersed throughout the epithelium produce a viscous mucus layer that covers the entire tract, except the large intestine, which is characterized by a double mucus layer. Similarly to mucus, mosquitoes secrete the peritrophic matrix (PM), a semi-permeable extracellular structure composed of chitin and glycoproteins. In larvae, the PM is secreted continuously by a specialized organ cardia located in the anterior midgut, whereas in adult females, it is believed to be secreted by the midgut epithelial cells only after blood feeding. Small **(A)** and large **(B)** intestines of mice harbor a series of gastrointestinal parasites: *Toxoplasma* exploits the small intestine for the establishment of the infection; *Giardia* and adult *Heligmosomoides polygyrus* worms thrive in the lumen of the small intestine, while adults of *Trichuris muris* anchor to the epithelial cells of the large intestine. In mosquitoes, *Plasmodium* invades the posterior midgut to establish extracellular infection at the basal side of the epithelial cells **(C)**. GL, gut lumen; CM, complex microbiota; ML, mucus layer; EC, epithelial cells; AC, abdominal cavity; PM, peritrophic matrix; BL, basal lamina; HL, hemolymph.

As early as 1885, Louis Pasteur postulated the preconceived idea that life under microorganism-free conditions would not be possible (Glimstedt, 1953). However, it was not until the 1950s that initial reports highlighted the importance of the symbiotic relationship between multicellular organisms and microorganisms, due to the generation of germ-free mammalian and insect animal models as novel research tools (Lancet, 1953). The complex communities of commensal bacteria, fungi, viruses and metazoans are collectively known as the microbiota. Importantly, the resident intestinal bacterial communities have been recognized as pivotal contributors to host development and metabolism, and for the induction and education of host immunity under homeostasis, during infection or inflammation (Grenham et al., 2011). Millions of years of evolution separate insects and mammals, which differ dramatically in many aspects, including the organization of their digestive and immune systems (Figure 1). However, they are often similarly exposed to the same microbes. Here we discuss how some of these commensal microbes affect immune responses of mice and mosquitoes to a number of parasites colonizing the intestinal tracts of their respective hosts.

In the context of immunity to gastrointestinal parasites, the microbiota of mammals is an essential functional player in the induction and maintenance of the mammalian immune system (Grainger et al., 2013). Removal of the resident gut microbiota inhibits the maturation of gut-associated lymphoid tissues, resulting in smaller and fewer Peyer's patches and lymphoid follicles (Cebra et al., 1998). From the initial colonization of the gut in neonates, the newly established commensal microbiota undergoes constant shifts in composition and diversity, reflective of the host's physiological development, diet, exposure to stress and gastrointestinal infections among other factors (Pickard et al., 2017). Mounting evidence has supported the notion that infection with intestinal parasites in mammals contributes to alterations in the diversity and abundance of commensal bacteria both locally and globally. On the other hand, insect vectors of medical importance like mosquitoes acquire and harbor a number of major human protozoan and nematode species in their own digestive tract. In recent years, stringent efforts have been placed to identify the factors that determine the mosquito vectorial capacity and to harvest that knowledge for the development of novel vector control strategies. Whereas, the midgut microbiota has emerged as a key factor shaping mosquito resistance to *Plasmodium*, the effector mechanisms driving these tripartite interactions remain largely unknown.

Although specific aspects of host-parasite-microbiota interactions have been recently reviewed separately for mice and mosquito vectors (Ippolito et al., 2018; Romoli and Gendrin, 2018; Stensvold and van der Giezen, 2018), in this review we contrast our current understanding of the relevance of microbiota-immunity interplay during gut infections in a comparative manner. We summarize and critically evaluate the role of the host intestinal microbiota in the instruction, support or suppression of mouse immunity to the non-invasive extracellular parasite *Giardia lamblia* and the invasive intracellular parasite *Toxoplasma gondii*, in comparison to the small intestinal nematode *Heligmosomoides polygyrus* and the large intestinal nematode *Trichuris muris*. We further discuss the most common insect effector mechanisms that control commensal and pathogenic bacteria, with a particular emphasis on their impact on *Plasmodium* infections in *Anopheles* mosquito vectors. Our review further highlights functional similarities and differences in microbiota-dependent induction and education of host immunity in such diverse hosts as mice and mosquitoes.

## Commensal microbiota composition of mice and mosquitoes

In the first year of life of most mammals, the microbial composition of their gastrointestinal tract develops from a sterile environment to one harboring a considerable density of commensal microorganisms, which over time develop a broad similarity to the microbiota composition of adults (Palmer et al., 2007). However, the infant microbiota presents considerably higher compositional variability and instability than the microbiota of adults, and factors like mode of delivery and breast feeding have been suggested as potential key factors in shaping the intestinal microbiota of mammals during the early stages of their life (Palmer et al., 2007; Milani et al., 2017; Pickard et al., 2017). Under homeostasis, the adult human intestinal tract harbors bacteria predominantly belonging to the *Bacteroides, Eubacterium, Ruminococcus*, and *Clostridium* genera, while the murine intestinal tract harbors predominantly *Clostridiales* and *Bacteroidales*, key in the enzymatic breakdown of complex polysaccharides (Palmer et al., 2007; Pickard et al., 2017). The high availability of mono and disaccharides in the gut, on the other hand, also allows for the proliferation of *Proteobacteria* and *Lactobacilli*–two other prominent members of the mammalian intestinal microbiota (Pickard et al., 2017). Notably, an abundance of evidence in recent years has highlighted that the normal composition of the adult intestinal microbiota suffers significant alterations following infections with gastrointestinal parasites, with significant implications to the regulation and maintenance of host metabolism and immunity.

Similar to mammals, the microbiota of *Anopheles* mosquitoes experiences dynamic changes in abundance and composition during the insect life cycle (Moll et al., 2001; Linenberg et al., 2016). The mosquito life cycle comprises four developmental stages: aquatic eggs, larvae, pupae and terrestrial adults. Larvae feed on environmental microorganisms, some of which establish residence in the larval guts and constitute the midgut microbiota. During pupariation, the resident microbiota is expelled together with the food bolus and the peritrophic matrix, resulting in dramatic losses of bacterial communities. Nevertheless, some larval bacteria can still be transmitted to pupae (Moll et al., 2001). In contrast, a stringent process of gut remodeling and sterilization during pupa-to-adult transition leads to complete loss of the pupal microbiome upon adult emergence (Moll et al., 2001). However, young adults re-establish microbial communities from bacteria-rich breeding water, whereby re-acquiring the microbial fingerprint of their larval environment (Lindh et al., 2008). Indeed, the diversity of the adult microbiota resembles the microbial composition of the aquatic larval habitats (Boissière et al., 2012; Gimonneau et al., 2014; Dickson et al., 2017). Larval diet shapes the microbial communities, impacts mosquito development and female susceptibility to infection with the human malaria parasite *Plasmodium* (Linenberg et al., 2016). In *Anopheles*, only female adults feed on blood to initiate their reproductive cycle. The change of diet from carbohydrate-rich nectar to protein- and lipid-rich blood induces massive bacterial proliferation and changes in microbial composition in the gut (Dong et al., 2009; Tchioffo et al., 2016). Despite the vast variability in the microbiota of individual mosquitoes from diverse geographical locations, γ*-Proteobacteria* dominate mosquito microbial communities and in particular, *Enterobacter, Serratia, Pantoea, Asaia, Aeromonas, Pseudomonas*, and *Bacillus* (Straif et al., 1998; Lindh et al., 2005; Rani et al., 2009; Boissière et al., 2012; Osei-Poku et al., 2012; Ngo et al., 2015).

## Intestinal parasite infections and associated changes in host microbiota

### Microbial changes induced by protozoan parasite infections

*Giardia lamblia* is an extracellular gastrointestinal parasite with a wide global distribution and still remains a common cause of food and waterborne-associated diarrhoeal disease (Figure 1A; Halliez and Buret, 2013). ***Giardia*** trophozoites attach to the epithelial lining of the small intestine and therefore they remain in intimate contact with the resident commensal microbiota of the host (Adam, 2001). *Giardia* infection in mice leads to localized shifts in the commensal microbial communities of the host small intestine. However, few studies have characterized the dynamics of *Giardia*-microbiota interactions in greater detail. During the early stages of infection, increased numbers of adherent and mucus-associated bacteria, as well as epithelial cell damage-related translocation of commensal bacteria from the intestine to the spleen and liver have been demonstrated (Chen et al., 2013; Halliez et al., 2016).

**Figure 2:**
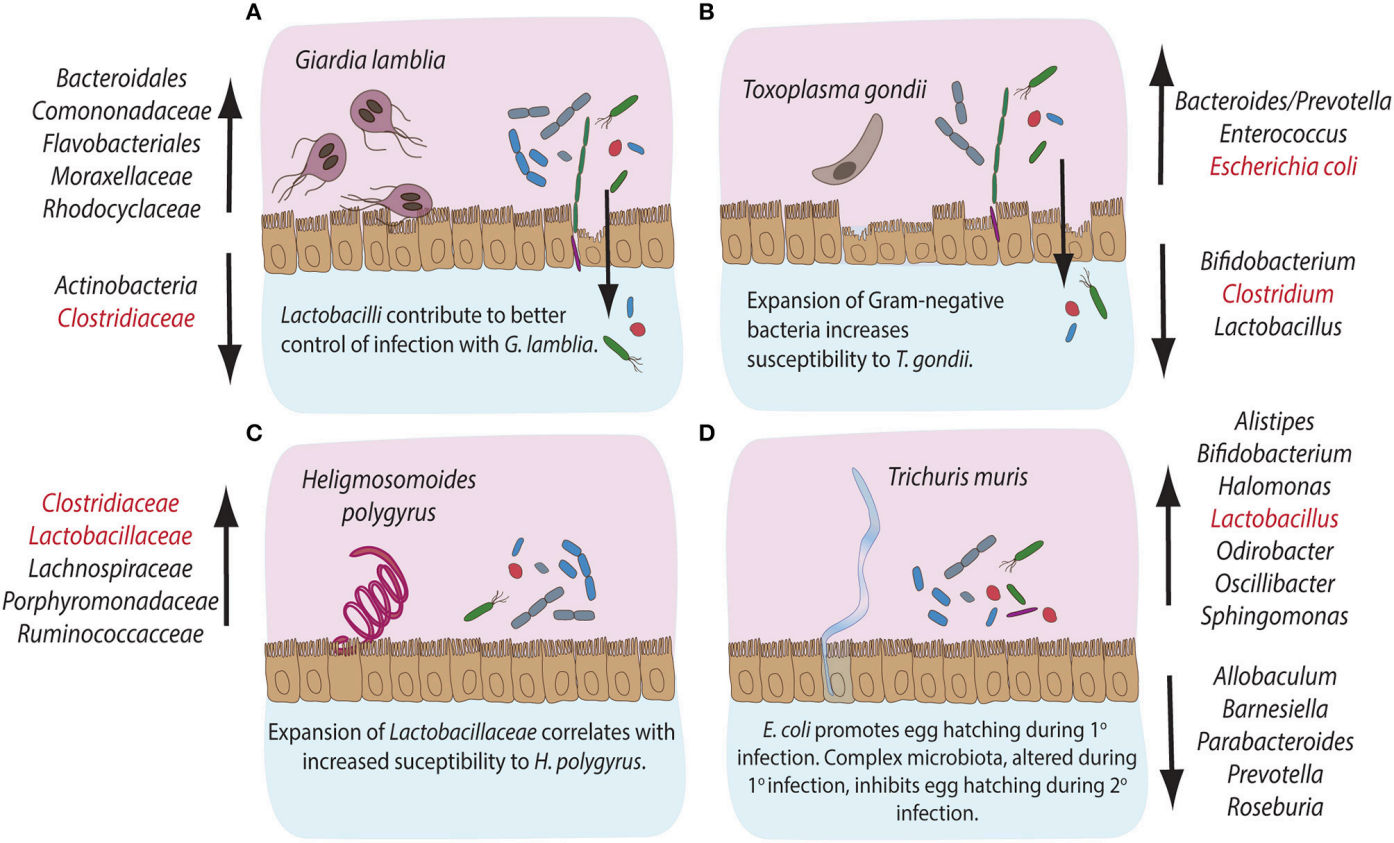
Compositional alterations and functional significances of commensal bacterial groups during intestinal parasite infections in mice. The gastrointestinal tract of mice is home to diverse communities of commensal bacterial species. Infections with intestinal pathogens are known to lead to notable alterations in the commensal microbiota composition of the host. **(A)** Infection with the extracellular protozoan parasite *Giardia lamblia* leads to a significant expansion of bacterial groups like *Bacteroidales, Comononadaceae, Flavobacteriales, Moraxellaceae* and *Rhodocyclaceae*, while *Actinobacteria* and *Clostridiaceae* display reduced abundances. On the other hand, *Lactobacilli* are known to contribute to an overall better control of *Giardia* infection. **(B)** The intracellular protozoan parasite *Toxoplasma gondii* leads to expansion in *Bacteroides/Prevotella, Enterococcus* and *Escherichia coli*, while the abundance of *Bifidobacteria, Clostridiales*, and *Lactobacilli* decreases during infection. An overall expansion of Gram-negative bacteria has been shown to contribute to immunopathology during murine infection with *T. gondii*. **(C)** Infection with the small intestinal parasitic nematode *Heligmosomoides polygyru*s leads to a marked expansion of commensal *Clostridiaceae, Lachnospiraceae, Porphyromonadaceae*, and *Ruminococcacce* and *Lactobacilli*, the latter known to correlate with increased susceptibility to infection with *H. polygyrus*. **(D)** Infection with the large intestinal nematode *Trichuris muris* leads to the expansion of *Alistipes, Bifidobacteria, Halomonas, Lactobacilli, Odirobacter, Oscillibacter*, and *Sphingomonas*, while *Allobaculum, Barnesiella, Parabacteroides, Prevotella*, and *Roseburia* abundances decrease. Notably, commensal *E. coli* is known to promote egg hatching during primary infection, while complex microbiota alterations occurring during primary infection appear to inhibit egg hatching during secondary infection with *T. muris*. The bacterial groups highlighted in red have been demonstrated to take part in induction, maintenance or suppression of host immune responses during the corresponding parasitic infections.

Acute infection with *G. lamblia* in mice has been shown to cause a significant expansion of β*-* and γ-*Proteobacteria* in the small intestine, cecum and colon, whereas the relative abundance of *Clostridia* and the diversity of *Melainabacteria* is decreased (Figure 2A; Barash and Maloney, 2017). Importantly, additional perturbations of the microbiota following antibiotic administration did not prevent the observed shifts in gut microbiota during murine giardiasis (Barash and Maloney, 2017). Investigations of the impact of diet on *Giardia*-induced changes in microbial composition have further revealed that while a low protein diet resulted in an increased *Firmicutes/Bacteroidetes* ratio, *Giardia* infection further enhanced the compositional shift in favor of *Firmicutes*, predominantly of *Clostridiales, Turicibacter*, and *Enterococcus* (Bartelt et al., 2017). In addition, *Giardia* infection increases excretion of bile acid derivatives, phosphatidylcholine, and taurine metabolites, as well as the availability of byproducts of glucose metabolism, indicating *Giardia*-induced alterations of host metabolism as a potential contributor to commensal microbiota alterations observed during infection (Barash and Maloney, 2017).

In contrast, the intracellular apicomplexan parasite *Toxoplasma gondii* initially infects the small intestine, where it induces significant immunopathological damage before quick systemic dissemination in the host (Figure 1A; Wilhelm and Yarovinsky, 2014). Despite the direct damage to host epithelial cells inflicted during intracellular parasite development, compositional changes in host intestinal microbiota have been established as a key factor driving intestinal immunopathology of toxoplasmosis in wild-type and humanized mice (Heimesaat et al., 2006; Bereswill et al., 2014; Von Klitzing et al., 2017). *T. gondii* infection causes a notable expansion of *Enterobacteriaceae, Bacteroides*, and *Enterococcus* (Heimesaat et al., 2006, 2014) and a decrease in *Lactobacillus, Bifidobacterium, Clostridia*, and *Bacteroidetes* species (Figure 2B; Heimesaat et al., 2006; Molloy et al., 2013). The expansion of predominantly Gram-negative commensal bacteria elevates proinflammatory cytokine production in the gut and the immunopathology of toxoplasmosis (Heimesaat et al., 2006). Similar to *Giardia, T. gondii* infection induces strong compositional changes in the gut microbiota, that contribute to the intestinal immunopathology of toxoplasmosis (Figure 2B).

### Microbial changes by intestinal nematode infections in mice

Infection with the murine small intestinal nematode *H. polygyrus* leads to profound changes in the host commensal microbiota along the entire gastrointestinal tract (Walk et al., 2010; Rausch et al., 2013; Reynolds et al., 2014). In particular, *Lachnospiraceae, Ruminoccoccaceae*, γ-*Proteobacteria/Enterobacteria*, and *Bacteroides* dominate the cecum, while a greater abundance of *Porphyromonadaceae, Lactobacillaceae*, and *Clostridiaceae* was detected in the ileum (Figure 2C; Walk et al., 2010; Rausch et al., 2013). Importantly, Reynolds et al. (2014) have correlated host susceptibility to infection with a significant increase in *Lactobacilli*. Overall, infections with *H. polygyrus* appear to change the balance between *Bacteroidetes* and *Firmicutes* in favor of the latter (Figure 2C).

The murine whipworm *T*. muris, on the other hand, develops in the large intestine, where the adult parasites protrude through the epithelium into the lumen (Holm et al., 2015). *Trichuris* adults, therefore, reside in close proximity the resident commensal microbiota of the host. Several studies have previously demonstrated that murine infection with *T. muris* results in shifts in the microbiota composition in the caecum and colon (Figure 2D; Holm et al., 2015; Houlden et al., 2015). During the early stages of infection, an increase in the abundance of *Bifidobacteria, Lactobacilli, Alistipes, Parabacteroides*, and *Odirobacter* has been observed. Later chronic stages increased the abundance of *Oscillibacter, Butyricicoccus, Parasutterella, Sphyngomonas*, and *Halomonas*, paralleled by a decrease in *Roseburia, Allobaculum*, and *Barnesiella* genera (Figure 2D; Holm et al., 2015). A significant reduction of *Bacteroides* correlated with a shift in the metabolite composition of the gut, affecting the biosynthesis of fatty acids, amino acids and phospholipids (Houlden et al., 2015). Interestingly, type 1 fimbriae adhesins, structural components of the commensal bacterium *E. coli*, were demonstrated to induce *T. muris* egg hatching *in vitro* (Hayes et al., 2010). Consistently, depletion of the host microbiota inhibits the establishment of infection *in vivo* (Hayes et al., 2010). Interestingly, changes in host microbiota caused by primary *T. muris* infection have been shown to inhibit egg hatching of secondary infections with *T. muris*, suggesting that some microbial products facilitate establishment of *T. muris* during primary, but not secondary infections (White et al., 2018). Overall, intestinal nematodes appear to change the microbiota composition of their hosts and alterations of the balance in abundances of *Firmicutes* and *Bacteroides* have been highlighted as a shared feature in these infection models.

### Microbial changes associated with *Plasmodium* infections in mosquitoes

In contrast to mammalian intestinal parasites residing primarily in the intestinal lumen and allow for continuous interaction between the parasites and the host microbiota, *Plasmodium* swiftly transits through the mosquito gut without establishing long-term residence in the lumen. The time that the parasite remains in contact with the luminal bacteria following a blood intake is limited to 24–30 h, by which time the majority of the parasites have already traversed the gut epithelium to escape the dangerous environment (Meis et al., 1989). Therefore, the impact of *Plasmodium* on the gut microbiota at this stage would be limited. Sporogonic parasite development at the basal side of the gut takes approximately 2 weeks. During this time, potential indirect parasite interactions with midgut bacteria could take place. Tchioffo et al. (2016) observed significant differences between mosquito microbial communities in the gut, ovaries and salivary glands at 1 and 8 days after *P. falciparum* infection. However, it is unclear whether immune or metabolic factors mediate these changes. Mosquito immune responses target predominantly the midgut-traversing ookinetes, whereas exposure of early oocysts to immune attacks is limited, questioning the role of anti-parasitic immunity in the observed microbiota changes (Garver et al., 2012). As *Plasmodium* oocysts scavenge mosquito lipids, and possibly other nutrients essential for their proliferation and virulence (Costa et al., 2017), the parasite may directly impact mosquito metabolism. However, it remains to be investigated whether *Plasmodium* competes with bacteria during its gut development for the same resources, or directly impacts mosquito metabolism.

## Micromanaging immunity

### Microbiota-associated immune responses to protozoan parasites

**Figure 3:**
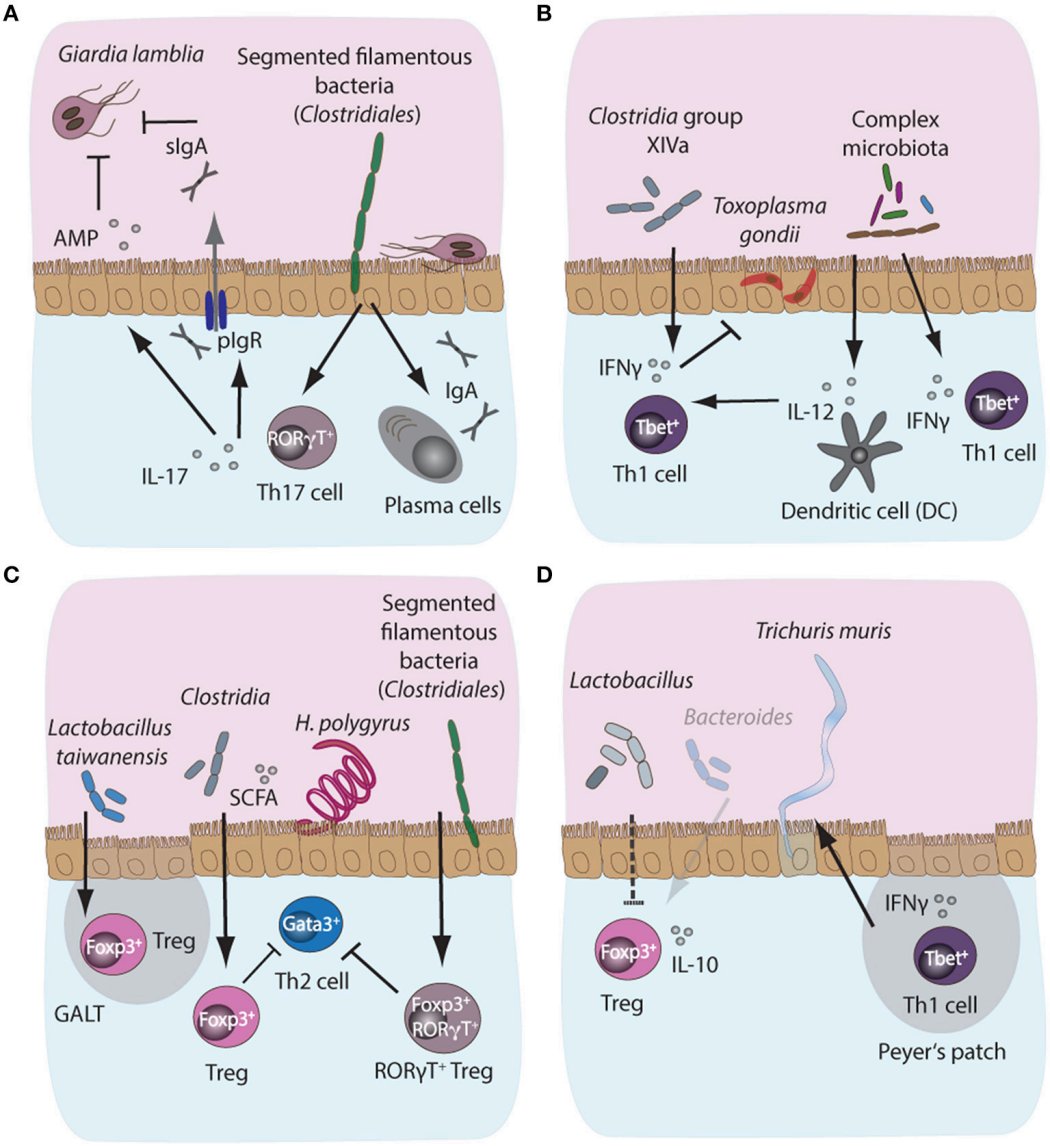
Microbiota-dependent host immune responses during intestinal parasite infections in mice. **(A)** Segmented filamentous bacteria (SFB), members of the *Clostridiales* bacterial group colonizing the small intestinal tract of mammals, are potent inducers of intestinal IgA and Th17 responses in mice. Importantly, both IgA and Th17 responses are key host protective immune mechanisms against the protozoan parasite *Giardia lamblia*. IgA can directly target trophozoites, while IL-17A supports polymeric Ig receptor expression and hence IgA transport into the lumen, in addition to promoting antimicrobial peptide (AMP) secretion by epithelial cells. Resistance phenotypes to giardiasis have previously been linked to the presence or absence of SFB, suggesting a potential correlation between microbiota-induced intestinal IgA and Th17 responses and control of infection with *Giardia*. **(B)** During acute murine infection with *Toxoplasma gondii*, the presence of complex intestinal microbiota leads to MyD88-dependent signaling, triggering protective IL-12 responses in intestinal dendritic cells. **(C)** During infection with the small intestinal nematode *Heligmosomoides polygyrus*, the increased abundance of *Lactobacilli* and *Clostridiales* producing short chain fatty acids (SCFA)have been shown to induce the expansion of Foxp3^+^ regulatory T cells (Treg) in the gut, while SFB additionally induce the development of a highly potent immunosuppressory subset of Foxp3^+^RORγT^+^ Treg, thus indirectly suppressing Th2 cell responses during infection. **(D)** A decrease in SCFA-producing *Bacteroides* and elevated abundances of *Lactobacilli* during chronic *T. muris* infection correlate with decreased intestinal Treg numbers and importantly, these Tregs appeared less prone to secrete IL-10 during chronicity.

Protective immunity to *Giardia* predominantly relies on the secretion of intestinal IgA and the induction of pro-inflammatory Th17 responses, supporting neutrophil recruitment and secretion of antimicrobial peptides (Dann et al., 2015; Saghaug et al., 2015). However, our current understanding of the potential immunomodulatory roles of the commensal microbiota in the induction and maintenance of host immunity against *Giardia* remains very limited. Although microbiota-independent CD4^+^ T-cell responses appear crucial for host protection, CD8^+^ T cell activation has been shown to be ablated in the gut of infected animals treated with antibiotics, suggesting a potential involvement of the intestinal microbiota in the activation of CD8^+^ T cell responses during giardiasis (Keselman et al., 2016). The mechanisms of CD8^+^ T-cell activation and their potential contribution to immune control during infection with *Giardia*, however, remain to be established (Keselman et al., 2016). Susceptibility to *G. lamblia* infection has previously been shown to vary in mice with an identical genetic background, but originating from independent commercial suppliers, further implicating host microbiota composition in shaping the course of *Giardia* infection (Singer and Nash, 2000). The microbiota of resistant mouse strains was later shown to contain Segmented Filamentous Bacteria (SFB), members of the family *Clostridiales*, while susceptible mice lacked this group (Ivanov et al., 2008). Importantly, SFB play a central role in the induction of intestinal Th17 responses (Ivanov et al., 2009), suggesting that microbiota-driven support for Th17 responses potentially facilitates immune control of *Giardia* infections. However, a direct link between the immunostimulatory properties of SFB and susceptibility to infection with *Giardia* remains to be established (Figure 3A).

*T. gondii* infection in mice, on the other hand, leads to potent Th1 immune responses and importantly, resident intestinal bacteria are known to play an important role in localized intestinal Th1 response polarization by providing molecular adjuvant signals in a TLR/MyD88-dependent manner, thus triggering IL-12 and IFN-γ production (Figure 3B; Benson et al., 2009). Studies have demonstrated the formation of distinct structured accumulations of host cells along the ileum of *T. gondii*-infected mice, named “intracellular casts,” which contained elevated levels of γ-*Proteobacteria* and were enriched for highly activated neutrophils and ROS-producing inflammatory monocytes (Grainger et al., 2013; Molloy et al., 2013). Further analysis revealed that the presence and expansion of γ-*Proteobacteria* positively influenced neutrophil infiltration into the lumen and, hence, played a role in the induction of intraluminal casts formation, suggesting that commensal bacteria potentially contribute to bacterial overgrowth and support the control of overt pathology during acute murine toxoplasmosis (Molloy et al., 2013). Additionally, inflammatory monocytes from small intestinal lamina propria were shown to adopt a mixed proinflammatory/regulatory phenotype during acute infection with *T. gondii*. The parallel secretion of IL-10, TNF-α, and PGE2 by these monocytes was demonstrated to depend on a range of commensal bacteria-derived ligands and has been correlated with potential PGE2-dependent suppression of commensal-driven neutrophil activation (Grainger et al., 2013). Moreover, toxoplasmosis is associated with the differentiation of CD4^+^ T cells specific for commensal microbes marked by a Th1 phenotype in a manner similar to parasite-specific Th1 cells (Figure 3B; Hand et al., 2012).

### Microbiota-associated immune responses to parasitic nematodes

Efficient immune control of intestinal nematodes largely depends on the development of protective type 2 immune responses driven by CD4^+^ Th2 cells in concert with type 2 innate lymphoid cells. Both cell types produce the Th2 cytokines IL-4, −5, and −13 leading to concerted immune effector mechanisms. These mechanisms fortify intestinal barriers via enhanced mucus production, increased epithelial cell turnover, intestinal fluid influx and hypercontraction of smooth muscle cells (Sorobetea et al., 2018). Nematode infections additionally support the activation and expansion of regulatory T cells (Tregs) (Maizels et al., 2012), a phenomenon linked to the anti-inflammatory effect of nematode infections in several models of autoimmunity (McSorley and Maizels, 2012).

Whether the microbiota affects the development of immune effector mechanisms during nematode infections has only recently received attention. One study has reported that low dose antibiotic treatment during *H. polygyrus* infection increased the abundance of members of the *Lactobacillaceae* and *Enterobacteriaceae* bacterial families (Reynolds et al., 2014). *Lactobacilli* abundance correlated positively with worm burdens, and, importantly, with higher numbers of Foxp3^+^ Tregs in gut-associated lymphoid tissue, suggesting *Lactobacilli*-mediated induction of Treg responses (Figure 3C). However, increased *Lactobacilli* abundance was observed only in C57BL/6 mice permissive for long-lasting infections with high worm burdens, while more resistant BALB/c mice did not display this phenotype. Interestingly, administration of *Lactobacilli* to more resistant mice increased their susceptibility to infection (Reynolds et al., 2014). In addition, *H. polygyrus-*dependent changes in intestinal microbiota composition have been correlated to the suppression of allergic airway inflammation. High concentrations of short-chain fatty acids (SCFA) resulted from the increased abundance of *Clostridiales* bacteria in *H. polygyrus* infected mice (Figure 3C; Zaiss et al., 2015). The SCFA increase supports mucosal Treg responses associated with the reduced susceptibility of mice to allergic airway inflammation (Zaiss et al., 2015). Furthermore, Ohnmacht et al. (2015) found that the microbiota is needed for the induction of highly activated RORγT^+^ Foxp3^+^ Tregs, which could be induced via the introduction of a cocktail of *Clostridia* species in germfree mice, most likely via the provision of the SCFA butyrate (Figure 3C). Importantly, the conditional removal of the RORγT^+^ Treg subset rendered *H. polygyrus*-infected mice more resistant to infection due to the development of a more robust Th2 response. Together, these observations suggest that the microbiota can indirectly control intestinal Th2 responses via the induction and maintenance of suppressive Treg subsets in the context of intestinal nematode infections (Figure 3C; Ohnmacht et al., 2015).

*T. muris* infection, on the other hand, leads to a notable decrease in *Bacteroides* producing SCFA, and a concomitant decrease in Foxp3^+^ Tregs in the lamina propria during chronic infection (Houlden et al., 2015). Moreover, Tregs in *T. muris* infection appeared less prone to release the anti-inflammatory cytokine IL-10. Therefore, chronically infected mice were more susceptible to intestinal inflammation and displayed poor worm expulsion due to the development of Th1 responses counteracting Th2 immunity (Figure 3D; Holm et al., 2015). Thus, in nematode infection, the commensal microbiota and, importantly, specific bacterial groups, appear essential for both the induction and suppression of host immune responses. Contextually, this can contribute to resistance or susceptibility to infection, depending on the bacterial community and the invading parasite species.

### Microbiota-dependent immune responses in mosquitoes

Multiple reports in the past have highlighted the ability of bacteria to inhibit the establishment of *Plasmodium* infection in the mosquito host (Gonzalez-Ceron et al., 2003; Dong et al., 2009; Cirimotich et al., 2011; Tchioffo et al., 2013; Bahia et al., 2014). Consistently, some antibiotic treatments promote parasite development (Beier et al., 1994; Dong et al., 2009; Gendrin et al., 2015, 2016). Plasmodicidal properties have been reported for diverse microbes and must rely on some general rather than species-specific mechanisms (Lowenberger et al., 1999; Gonzalez-Ceron et al., 2003; Dong et al., 2009; Cirimotich et al., 2011; Tchioffo et al., 2013).

**Figure 4:**
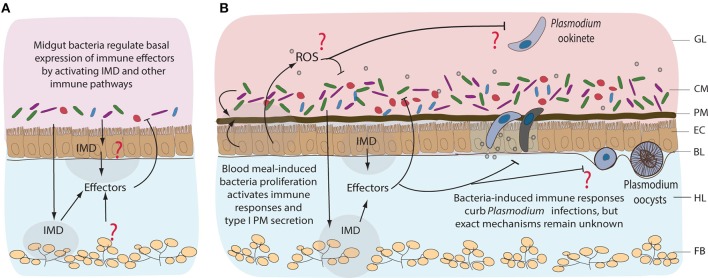
Immune responses of mosquitoes to midgut bacteria and to *Plasmodium* parasites. Immune responses to resident bacteria before **(A)** and after blood feeding **(B)**. Proliferation of the midgut bacteria is regulated mainly by IMD pathway, however the tissue specificity of its expression remains unknown. Immune responses could be coming from the gut, hemocytes (mosquito blood cells), or fat body (nutrient storage and immune tissue). Involvement of other immune pathways, including reactive oxygen species (ROS), in response to midgut bacteria is not well-characterized. Blood feeding-induced bacteria proliferation activates immune responses that also curb the invasion of *Plasmodium*, mainly at the ookinete stage of development. GL, gut lumen; CM, complex microbiota; PM, peritrophic matrix; EC, epithelial cells; BL, basal lamina; HL, hemolymph; FB, fat body.

Female mosquitoes alternate between sugar and blood feeding. Such diverse diets expose the gut to strong physiological and oxidative challenges (reviewed in Sterkel et al., 2017). Uptake of proteins and lipids from iron and heme-rich blood induces massive metabolic and transcriptional changes in the midgut (Figure 4). Nutrient abundance sets off bacterial proliferation which, in turn, induces expression of immune genes (Dong et al., 2009). Blood intake elevates hydrogen-peroxide levels in the mosquito hemolymph and activates reactive oxygen species (ROS) detoxification responses (Kumar et al., 2003; Molina-Cruz et al., 2008; de Almeida Oliveira et al., 2012). However, how blood-feeding affects ROS levels in the midgut, and the underlying mechanisms remain unknown (Figure 4B). What is clear is that ROS inhibition after blood feeding causes lethal systemic bacterial infections, suggesting a crucial link between oxidative stress and immune activation (Molina-Cruz et al., 2008).

The peritrophic matrix (PM) provides the first line of defense against blood meal-induced oxidative stress and bacterial infections (Figures 1, 4B). The cardia cells of mosquito larvae continuously secrete sleve-shaped PM (type II), whereas adult epithelial midgut cells synthesize type I PM only after blood feeding (Wigglesworth, 1930; Waterhouse, 1953). Feeding on complex organic matter continuously exposes larvae to bacteria. In contrast, blood feeding induces massive bacterial proliferation in the gut and renders adult females particularly vulnerable to microbial infections (Dong et al., 2009; Linenberg et al., 2016). Such differences in mosquito exposure to microbes at these developmental stages likely contribute to constant PM secretion in larvae vs. inducible PM secretion in adults. Interestingly, PM synthesis in adults requires midgut microbiota. Bacterial proliferation after blood feeding upregulates expression of hundreds of genes, including the genes encoding PM proteins such as glucosamine-fructose-6-phosphate aminotransferase (GFAT) and chitin synthase enzyme 1 (CHS1; Rodgers et al., 2017). Conversely, antibiotic treatment before blood feeding compromises the integrity of PM by inhibiting expression of *GFAT* and *CHS1*, but also the genes encoding peritrophic proteins 1 and 14, whereas it upregulates expression of the PM-degradation chitinase genes A and B (Rodgers et al., 2017; Song et al., 2018). The PM of mosquitoes, therefore, serves as an inducible protective mechanical barrier against bacteria.

The immune-deficiency (IMD) pathway, initially identified in *Drosophila*, is the major immune pathway that control bacterial infections by coordinated expression of antimicrobial peptide (AMPs) genes (Buchon et al., 2009; Broderick et al., 2014). In *Anopheles*, the pathway is activated by the recognition of DAP-type and lysine-type peptidoglycans by the peptidoglycan recognition protein LC (PGRP-LC; Meister et al., *2009)*. Although relatively understudied in the mosquito gut, several lines of evidence suggest that IMD is functional in this tissue. *A. gambiae* mosquitoes with intact microbiota display higher basal expression levels of immune genes compared to antibiotic-treated controls (Figure 4A; Dong et al., 2009). These genes encode a range of AMPs, signal-transducing serine proteases, IMD pathway components and immune genes such as fibrinogen-related and thioester-containing proteins among others (Dong et al., 2009). Importantly, silencing of a transcriptional activator of the NF-kB family, *REL2* and of *PGRP*-*LC* receptor, promotes the proliferation of gut microbiota and increases mosquito susceptibility to *Plasmodium* infection (Dong et al., 2009; Meister et al., 2009). The proliferation of bacteria is believed to activate the IMD pathway via the PGRP-LC receptor. Since bacterial proliferation 24 h after blood feeding coincides with ookinete transversal of the midgut epithelium, it may also lead to the IMD-mediated killing of *Plasmodium* parasites (Figure 4B; Dong et al., 2009; Meister et al., 2009; Linenberg et al., 2016). Indeed, clearance of the mosquito microbiota by antibiotics before infection increases mosquito susceptibility to *Plasmodium*, whereas bacteria inoculation by feeding decreases it in a PGRP-LC-dependent manner (Meister et al., 2009).

*Wolbachia*, intracellular maternally transmitted bacteria, confer protection to their arthropod hosts against a range of pathogens (Hedges et al., 2008; Teixeira et al., 2008; Kambris et al., 2009; Moreira et al., 2009; Hughes et al., 2011). *Wolbachia* has been identified in natural *Anopheles* populations in Burkina Faso and Mali (Baldini et al., 2014; Gomes et al., 2017). Importantly, *Wolbachia* infections (experimental transovarian of the Asian malaria vector *A. stephensi*, natural infections of *A. coluzzii* and somatic infections in *A. gambiae*) significantly decrease the prevalence of *P. falciparum-*infected mosquitoes (Hughes et al., 2011; Bian et al., 2013; Shaw et al., 2016; Gomes et al., 2017). Although the mechanisms that cause mosquito resistance to *Plasmodium* remain to be elucidated, experimental *Wolbachia* infections induce expression of a series of immune effectors and ROS which could potentiate parasite killing (Kambris et al., 2009; Bian et al., 2013).

In addition to inducing the immunity-mediated *Plasmodium* killing, some bacteria show direct plasmodicidal activity. *Enterobacter* isolates from Zambian *Anopheles* kill *P. falciparum* in the midgut when administrated with the infectious blood meal (Cirimotich et al., 2011). This has been attributed to direct inhibition of ookinete development by the bacteria-produced ROS (Cirimotich et al., 2011). Soluble factors released by *Serratia marcescens* also inhibit *P. falciparum* ookinete development in the mosquito (Bahia et al., 2014). Inhibition of *P. berghei* ookinetes *in vivo* was linked to the length of the flagella and the motility of *S. marcescens* (Bando et al., 2013). Currently, it is unclear whether this inhibition is direct or immunity-mediated. Furthermore, oral administration of *Chromobacterium* sp. isolated from *A. aegypti* in Panama, induces high mortality in mosquito larva and adults, whereas the surviving mosquitoes exhibit low *Plasmodium* infection loads (Ramirez et al., 2014). Biochemical analyses identified romidepsin, the histone deacetylase inhibitor, as the plasmodicidal factor, uncovering an essential role of histone modifications in the mosquito stages of *Plasmodium*. Surprisingly, administration of romidepsin did not affect mosquito survival, suggesting that another bacterial factor mediates the *Chromobacterium*-induced mortality (Saraiva et al., 2018).

How plasmodicidal properties of mosquito microbiota correlate with dynamics of *Plasmodium* transmission in the field remains unknown. In laboratory conditions, *Serratia sp*. and *Enterobacteriaceae* curb *Plasmodium* infection (Bando et al., 2013; Tchioffo et al., 2013; Bahia et al., 2014). In contrast, two semi-field studies in Cameroon associated the same bacteria with higher *P. falciparum* loads (Boissière et al., 2012; Tchioffo et al., 2016). Most of the experimental studies examined the role of microbes in *Plasmodium* infections using microbiota-free mosquitoes, and the results of the few field studies stress the importance of studying the tripartite interactions between mosquitoes, microbes and parasites in natural conditions.

## Bacterial feeding vs. bacterial clearance

### Manipulating host microbiota as a treatment strategy against protozoan parasites

Treatment of giardiasis primarily relies on the administration of antibiotics like metronidazole. However, due to rising levels of resistance, frequent reports of side effects and clinical failures, there is a growing need for the development of novel alternative treatment strategies (Table 1; Ansell et al., 2015). In the past, it has been shown that the use of *Lactobacilli* as probiotics in the treatment of giardiasis holds promise. In *G. duodenalis*-infected gerbils, administration of *L. johnsonii La1* leads to reduced infection rates, lack of pathological damage to the epithelial cell layer and absence of immune cell infiltration and inflammation in mice receiving the probiotic compared to the placebo group (Humen et al., 2005). One proposed mechanism of the observed anti-giardial activities of *L. johnsonii La1* has been attributed to the metabolic generation of unconjugated bile salts (Pérez et al., 2001; Travers et al., 2016). Very recently, Allain et al. tested *in vitro* and *in vivo* the anti-giardial activity of three bile salt hydrolases (BSH), enzymes naturally produced by *L. johnsonii La1* (Allain et al., 2018). *In vitro* treatment of *G. duodenalis* with increasing concentrations of recombinant BSH47 and BSH56 enzymes revealed a dose-dependent giardicidal activity of both enzymes in the presence of bile. Importantly, the group further demonstrated the anti-giardial activity of rBSH47 after administration to *G. duodenalis*-infected suckling mice, supporting the view of *L. johnsonii La1* as a probiotic candidate for the treatment of *Giardia* infections.

**Table 1:** Administration modes, outcomes and proposed mechanisms of action of probiotic bacteria as potential treatment strategies against intestinal parasitic infections in murine hosts and mosquito vectors.

**Parasite species**	**Host species**	**Commensal bacterial group**	**Treatment**	**Outcome**	**Proposed mechanism of action**	**References**
*Giardia lamblia*	*Meriones unguiculatus Mus musculus*	*Lactobacillus johnsonii* La1 (NC533)	*Ad libidum* administration of 10^8^ CFU per animal 7 days prior to infection	Reduced infection rates, lack of epithelial cell layer damage, no immune cell infiltration and lower inflammation rates (compared to placebo-treated control animals)	Metabolic generation of bile salts with direct anti-giardicidal properties	Pérez et al., 2001; Humen et al., 2005; Travers et al., 2016; Allain et al., 2018;
	*Mus musculus*	*Enterococcus faecalis* SF68	*Ad libidum* administration of 5 × 10^8^-1 × 10^9^ CFU continuously during trial, starting 7 days prior to infection	Elevated total IgA levels in intestine, as well as higher titers of intestinal *Giardia*-specific IgA and serum IgG, increased proportion of CD4^+^ T-cells in spleen and Peyer's patches	Induction of naturally polyreactive intestinal IgA secretion	Benyacoub et al., 2005
*Toxoplasma gondii*	*Mus musculus*	*Bifidobacterium animalis* subsp. *lactis*	*Ad libidum* administration of 1.6 × 10^7^ CFU suspended in 0.1 mL milk continuously during trial, starting 15 days prior to infection	Elevated *Toxoplasma*-specific IgG in serum, elevated numbers of CD19^+^ B-cells, reduced brain cyst numbers and lower intestinal villi inflammation	Induction of protective humoral immune responses during infection	Ribeiro et al., 2016
*Trichuris muris*	*Mus musculus*	*Lactobacillus casei* (ATCC7469)	*Ad libidum* administration of live or dead 1.8 × 10^9^ CFU 7 days prior to infection	Suppression of localized IFN-γ and TNF-α responses in secondary lymphoid organs, elevated worm burdens	Unknown	Dea-Ayuela et al., 2008
	*Mus musculus*	*Lactobacillus rhamnosus* JB-1	*Ad libidum* administration of 1 × 10^9^ CFU for 15 days or 36 days, starting 1 day prior to infection	Elevated tissue levels of IL-10, higher numbers of goblet cells and accelerated worm expulsion	Induction of protective IL-10 production by epithelial cells and enhanced mucus production during infection	McClemens et al., 2013
*Plasmodium falciparum*	*A. gambiae A. stephensi*	*Wolbachia*	Embryonic microinjection	Somatic infection, reduced prevalence and intensity of infection in *A. gambiae* Somatic and ovarian infection, invasion of *A. stephensi* populations, reduced infection rates	Unknown. Potentially through activation of immune defenses	Hughes et al., 2011; Bian et al., 2013
	*A. gambiae*	*Enterobacter*	Administration of 10^3^–10^5^ CFUs with the infectious blood meal	Inhibition of *Plasmodium* ookinete stage, reduced infection rates	Bacteria-produced ROS	Cirimotich et al., 2011
	*A. gambiae*	*Serratia marcescens*	10^3^–10^7^ bacteria/μl introduced to aseptic mosquitoes with the blood meal	Reduced infection rates and reduced survival after blood feeding	Unknown. Potentially through inhibition of *Plasmodium* ookinetes by *Serratia* soluble factors or bacterial flagellum (both demonstrated with *P. berghei*)	Bando et al., 2013; Bahia et al., 2014
	*A. gambiae*	Engineered *Serratia marcescens* expressing anti-malarial effector proteins	10^7^ bacteria/ml introduced via sugar meal	Colonized midgut and reproductive organs, transmitted in three successive generations. Reduced infection rates	Through secretion of anti-*Plasmodium* effector molecules	Wang et al., 2017

Along the same line, *Enterococcus faecalis* SF68, a lactic acid bacterium indigenous to the mammalian commensal microbiota, has also been suggested as a potential probiotic treatment for *Giardia*. Oral administration of this strain prior to *G. lamblia* infection led to elevated levels of total IgA levels in small intestine during the acute stage of infection, as well as higher levels of specific anti-*Giardia* IgA and systemic IgG compared to control animals. Nevertheless, parasitological data were less conclusive and the use of *E. faecalis* SF68 as a probiotic supporting anti-*Giardia* immunity merits further investigations (Benyacoub et al., 2005). Overall, it appears that administration of probiotic *Lactobacilli* in *Giardia*-infected mice reduces epithelial damage, attenuates overt inflammation and fosters enzymatic giardicidal activity.

Treatment of toxoplasmosis, on the other hand, relies primarily on the administration of pyrimethamine and sulfonamide drugs (Alday and Doggett, 2017). Recently, the administration of *Bifidobacterium animalis* subsp. *lactis* to mice chronically infected with *T. gondii* demonstrated enhanced serum levels of anti-*T. gondii* IgG and higher numbers of CD19^+^ B-lymphocytes, a reduction in brain cysts, and attenuated intestinal villi inflammation in the probiotic-treated group (Ribeiro et al., 2016). Conversely, previous work in gnotobiotic mice colonized by probiotic *E. coli* strains revealed that these microbes contribute significantly to intestinal inflammation during *T. gondii-*infection (Bereswill et al., 2013). These results indicate that the use of probiotic bacterial strains in the treatment of intestinal parasite infections is highly contextual and reports on the potential benefits and risks for host health should be considered in the future design of novel probiotics.

### Probiotic administration and microbiota manipulation during nematode infections

In intestinal helminth infections, few *in vivo* or *in vitro* studies have demonstrated mechanisms of action of probiotic bacteria favoring either infection resistance or susceptibility. Treatment of *T. muris*-infected mice with viable or dead probiotic *Lactobacillus casei* led to the maintainance of high worm burdens in the chronic phase of infection and concurrently suppressed cellular and Th1/Th2 cytokine responses in secondary lymphoid organs (Dea-Ayuela et al., 2008). On the other hand, administration of live *Lactobacillus rhamnosus* JB-1 to *T. muris-*infected mice resulted in accelerated worm expulsion and elevated gut tissue concentrations of IL-10, but not Th2 or Th1 cytokines. Administration of *L. rhamnosus* enhanced worm expulsion in mice deficient in mucin 2 (Muc2^−/−^), a key component of the intestinal mucus layer led to elevated goblet cell frequencies compared to medium-treated Muc2^−/−^ mice, suggestive of probiotic-dependent induction of additional mucins involved in worm expulsion (McClemens et al., 2013).

The administration of probiotic bacteria against gastrointestinal protozoan parasite infections has gained attention as a novel and effective treatment strategy (Table 1). Numerous studies have highlighted a range of beneficial aspects of probiotic bacteria including support for intestinal antibody responses, reduction in immunopathology and elevated anti-inflammatory cytokine responses, as well as potential competition for nutrients with the invading intestinal parasite species. On the other hand, the introduction of certain probiotic bacteria during intestinal parasite infections could also have a number of detrimental effects, including increasing the numbers of intestinal Tregs and thus potentially facilitating nematode survival or providing support for damaging inflammatory reactions during infection.

### Microbiota manipulations in mosquitoes as a vector control strategy

The environmental bias of the mosquito microbiome poses a significant challenge for its manipulation for vector control purposes. However, several approaches have been explored for introducing into natural mosquito populations bacteria with anti-*Plasmodium* properties (Table 1). *Wolbachia* has been successfully exploited in *A. aegypti* mosquitoes to interrupt the transmission of dengue virus (Hoffmann et al., 2011, 2014). Efficient *Wolbachia* application for malaria control requires rapid bacterial spread in the mosquito populations. This spread relies on *Wolbachia* ability to induce cytoplasmic incompatibility (CI) in the host, which is manifested by the sterility of individuals with different *Wolbachia* infection status (Bordenstein and Werren, 2007). CI has not been observed in *Wolbachia* naturally occurring in *A. gambiae*. Instead, the bacteria appear to induce a modest acceleration of egg-laying rates (Shaw et al., 2016). For experimentally-introduced *Wolbachia*, successful maternal transmission and CI has been reported in *A. stephensi* (Bian et al., 2013). Another study has shown inhibition of vertical *Wolbachia* transmission in *A. gambiae* and *A. stephensi* by resident microbiota (Hughes et al., 2014), while other attempts to stably introduce *Wolbachia* in *A. gambiae* have been unsuccessful (Kambris et al., 2009; Hughes et al., 2011).

Recently, mosquito colonization with genetically engineered *Serratia* has been proposed as an alternative transmission-blocking strategy (Wang et al., 2017). In addition to the plasmodicidal activity of *S. marcescence* described above, the engineered *Serratia* strains express one or multiple anti-malarial effector proteins and successfully decrease *P. falciparum* infections in *A. stephensi* (Wang et al., 2017). *Serratia* naturally colonizes the mosquito midgut, ovaries and male accessory glands, and is expected to spread throughout mosquito populations. However, the persistence of genetically engineered *Serratia* under laboratory conditions was only demonstrated for three successive generations with a substantial drop in bacterial loads already in the second generation (Wang et al., 2017).

A surprising link between immune activation and changes in microbiota composition of the mosquito reproductive organs has been reported recently. Transgenic mosquitoes that expressed an active form of the REL2 transcriptional factor in the midgut after a blood meal were reported to inhibit bacterial proliferation and *Plasmodium* invasion (Dong et al., 2011). Unexpectedly, activation of the IMD pathway modified the mosquito mating behavior leading to the preferential mating of transgenic males with wild-type females (Pike et al., 2017). Although the exact mechanism is unclear, the authors proposed that transgene expression inhibits bacterial proliferation in the male reproductive organs and facilitates the spread of the transgene (Pike et al., 2017). Surprisingly, female mating preference was not affected by the transgene expression. Whether such behavioral manipulation was caused by a particular bacterial species or the overall bacterial loads and whether transgenesis-induced changes were male-specific remains to be investigated.

Another interesting approach to mosquito control exploits the entomopathogenic bacteria *Chromobacterium* sp. and the fungus *Beauveria bassiana* that exhibit plasmodicidal activities. As discussed above, *Chromobacterium* interferes with *Plasmodium* through histone modification processes and induces mortality by an as yet unknown mechanism (Saraiva et al., 2018). *B. bassiana*, on the other hand, downregulates IMD-mediated immunity and expression of dual oxidase DUOX, causing midgut dysbiosis and systemic infections by opportunistic bacteria that kill mosquitoes (Wei et al., 2017). Therefore, disrupting or manipulating the mosquito microbiome often has fatal consequences for the vector that may directly or indirectly contribute to the inhibition of *Plasmodium* development.

## Conclusions and future perspectives

Along the entire length of the mammalian intestinal tract from the small intestine infected by *Giardia, T. gondii*, and *H. polygyrus*, to the large intestine where *T. muris* thrives, there is evidence of a critical and vastly complex relationship between the host immune system, the resident microbiota and the invading pathogens. Alterations of the host microbiota can both affect the integrity and efficiency of protective immune responses, as well as the development of the parasite and its infection efficacy. However, we still lack a more comprehensive understanding of the causality vs. correlation between specific compositional shifts of the commensal bacterial communities in the gut and the induction of host immune mechanisms during intestinal parasite infections. More specifically, the majority of current studies are performed in laboratory conditions, often with microbiota-free animals. Despite their limited sample size, field and semi-field studies provide valuable insights into the microbiome complexity of natural *Anopheles* populations and the effects of natural microbial communities on *Anopheles* vectorial capacity and anti-parasitic responses in mice deserve further investigation.

The intestinal microbiota of mice and mosquitoes naturally faces vastly contrasting environmental and biochemical challenges due to major differences in host development, physiology and diet among many other factors (Table 2). The mouse microbiota displays higher complexity and species diversity than the resident microbial community in the mosquito, but at the same time experiences less drastic variations and expansion compared to those seen in freshly blood-fed mosquito females. While the significant expansion of γ*-Proteobacteria* documented during acute *T. gondii* infection in mice contributes to immunopathology due to the activation of neutrophils and ROS-producing inflammatory monocytes, γ*-Proteobacteria* are important players in limiting vector competence of mosquitoes for *Plasmodium*. More importantly, the generation of ROS is a crucial immune response mechanism in insects, induced in response to a range of microorganisms. Considering the importance of murine resident γ*-Proteobacteria* in the induction of ROS-producing inflammatory monocytes against *T. gondii*, it merits further investigations whether a similar bacteria-mediated process is limiting vector capacity of mosquitos after uptake of *Plasmodium* with the blood meal.

**Table 2:** Comparison of intestinal immunity, microbial diversity and microbiota-related interactions within the murine hosts and the mosquito vectors during intestinal parasitic infections.

**Comparison**	**Mouse**	**Mosquito**
Mechanical barriers	Mucus layer, enriched in mucin glycoproteins, permanently lining the intestinal epithelial layer as a shield from direct exposure to external stimuli and invading microorganisms. Commensal microbiota supports mucus secretion and maintenance.	Semipermeable peritrophic matrix (PM) composed of chitin and glycoproteins, lining the entire larval gut. In adult females, PM is secreted in the midgut upon blood feeding, which induces significant microbial proliferation in the midgut. Commensal microbiota stimulates peritrophic matrix synthesis.
Intestinal microbial diversity	Higher complexity and species diversity. Compositional changes occur subject to environmental and dietary changes like breastfeeding or gastrointestinal infections. Exhibits overall less drastic compositional shifts during lifespan of host. Dominant bacterial groups include *Enterobacter, Lactobacillus, Bifidobacteria, Bacteroides, Clostridia, Ruminococcus*.	Lower complexity and species diversity. High fluctuation in diversity and species abundance observed throughout the development and after blood meal uptake. Exhibits drastic compositional shifts during vector development. Dominant bacterial groups include *Enterobacter, Serratia, Pantoea, Asaia, Aeromonas, Pseudomonas, Bacillus*.
Microbial fingerprint during parasitic infections	Numerous studies have demonstrated significant alterations of intestinal microbial communities during infections with protozoan parasites (eg. *Giardia lamblia* and *Toxoplasma gondii*), as well as during nematode infections with *Heligmosomoides polygyrus* or *Trichuris muris*.	Not enough evidence of *Plasmodium* influence on midgut microbial communities, possibly due to limited time spent by the parasite in the midgut lumen.
Microbial priming of immune defenses against parasitic infections	Segmented filamentous bacteria (family *Clostridiales*) are known potent inducers of intestinal Th17 responses, key for host protection against *Giardia* infection.	Midgut microbiota is a potent inducer of IMD/NF-κB pathway. Basal activity of IMD is regulated by the midgut microbiome, and bacterial proliferation after blood feeding further enhances pathway activation. The microbiota-dependent activation of the IMD pathway impacts within-mosquito development of *Plasmodium falciparum*.
	*Lactobacilli* induce intestinal Treg responses during infection with *H. polygyrus*, but not during *T. muris* infection, and correlate with higher worm burdens	*Enterobacter* isolates have direct plasmodicidal activity. They produce ROS that kills *Plasmodium* ookinetes. Soluble factors of *Serratia marcescens* and motility of some *Serratia* strains impact development of *P. berghei* ookinetes.
	*Clostridia* can induce suppressive Treg responses via short-chain fatty acid production during *H. polygyrus* infection	Although understudied, immune activation after blood feeding is linked to oxidative stress. Blood feeding increases hydrogen peroxide levels and ROS detoxification responses. Moreover, inhibition of ROS leads to lethal systemic bacterial infections.
	γ*-Proteobacteria*, among the most abundant commensal bacteria in mice, contribute to intestinal immunopathology during *T. gondii* infection via activation of neutrophils and ROS-producing inflammatory monocytes	γ*-Proteobacteria* is the most abundant class of bacteria in mosquitoes. It limits mosquito vector competence for *Plasmodium* transmission by inducing the IMD pathway and probably other as yet unknown mechanisms.

Following each blood meal, the mosquito midgut synthesizes a semipermeable peritrophic matrix that shields the gut epithelium from direct exposure to potentially harmful external stimuli and invading microorganisms. Similarly, the murine gastrointestinal tract contains a viscous layer of mucus enriched in mucin glycoproteins protecting the intestinal epithelial layer (Table 2). Microbes enhance mucus secretion in the mammalian gut similarly to what has been suggested for PM deposition in the mosquito. Further studies are required to see if distinct microbes support the peritrophic membrane and thereby limit *Plasmodium* infection.

Changes in microbiota induced during intestinal parasite infections may provide valuable information on new intervention strategies against these pathogens. A better mechanistic understanding of how the microbial status and infection-induced microbiome alterations of an individual affects immune responses to parasites and of how the microbiome could be targeted to reduce infection success of parasites and arthropod vectorial capacity necessitates further studies. So far, the diversity in study designs using a gnotobiotic, mono-colonized, antibiotic-treated or standard-pathogen-free mouse and vector models offers invaluable tools for the elucidation of effector mechanisms responsible for the induction and maintenance of immune responses and to what extent specific members of the commensal microbiota are involved. Nevertheless, variations in study design like the colonization status of the host/vector, host age, sex, commercial provider, parasite strain and infection dosage, microbiota culturing and sampling techniques, as well as differences in data analysis at the phylum, order, family, or genus level present considerable challenges to microbiota research. Caution must also be taken in the interpretation of studies on microbiota-dependent immunity and the translation of their findings outside the laboratory, as inbred lab-reared mice/vectors experienced a considerable change in bacterial diversity/composition compared to wild animals, which provide a more representable animal model. Future studies should consider parallel investigations of wild rodent models and vectors from natural populations for a more comprehensive understanding of the tripartite interactions between host, microbiota and invading intestinal parasites. The administration of cocktails of probiotic bacteria with varying stimulatory effector functions, rather than single bacterial groups would represent successful future probiotic treatment strategies. As such, further studies focusing on how commensal and probiotic bacterial communities communicate and regulate each other in the context of gastrointestinal infections would be required in humans, laboratory and free-living mice and mosquito species alike.

## Author contributions

All authors contributed to the work presented in this manuscript. EL and SH conceived the original framework of this paper. IY and SZ performed the literature search and wrote the manuscript. EL, SR, GC, and SH edited and critically revised the manuscript.

### Conflict of interest statement

The authors declare that the research was conducted in the absence of any commercial or financial relationships that could be construed as a potential conflict of interest.
